# How Valuable Are Small Measurement Datasets in Supplementing Occupational Exposure Models? A Numerical Study Using the Advanced Reach Tool

**DOI:** 10.3390/ijerph20075386

**Published:** 2023-04-04

**Authors:** Kevin McNally

**Affiliations:** HSE Science and Research Centre, Health and Safety Executive, Buxton SK17 9JN, UK; kevin.mcnally@hse.gov.uk

**Keywords:** Bayesian exposure assessment, occupational exposure, chemical regulation, validation

## Abstract

The Advanced REACH Tool (ART) is the most detailed exposure model currently available for estimating inhalation exposures to dusts, vapours, and aerosols under a broad range of exposure scenarios. The ART follows a Bayesian approach, making use of a calibrated source–receptor model to provide central estimates of exposures and information on exposure variability from meta-analyses in the literature. Uniquely amongst exposure models, the ART provides a facility to update the baseline estimates from the mechanistic model and variance components using measurement data collected on the exposure scenario; however, in practical use, this facility is little used. In this paper, the full capability of the ART tool is demonstrated using a small number of carefully chosen case studies that each had a sufficient breadth of personal exposure measurement data to support a measurement-led exposure assessment. In total, six cases studies are documented, three where the estimate from the source–receptor model of the ART was consistent with measurement data, and a further three case studies where the source–receptor model of the ART was inconsistent with measurement data, resulting in a prior-data conflict. A simulation study was designed that involved drawing subsets of between two and ten measurements from the available measurement dataset, with estimates of the geometric mean (GM) and 90th percentile of exposures from the posterior distribution of ART compared against measurement-based estimates of these summaries. Results from this work indicate that very substantial reductions in the uncertainty associated with estimates of the GM and 90th percentile could be achieved with as few as two measurements, with results in detail sensitive to both the measurements themselves and worker and company labels associated with the measurements. For case studies involving prior-data conflicts, the estimates of the GM and 90th percentile rapidly changed as measurement data were used to update the prior. However, results suggest that the current statistical model of the ART does not allow a complete resolution of a prior-data conflict.

## 1. Introduction

Datasets of representative exposure measurements are widely considered to provide the gold standard for estimating inhalation exposures to workers in the occupational setting. The guidance for supporting occupational exposure assessments using measurement data under the Registration, Evaluation, Authorization and Restriction of Chemicals (REACH) legislation is documented in part R.14 of the guidance document [1]. The guidance document notes that a measured dataset supporting an exposure scenario must describe the conditions of use at a specific site or a range of very similar sites and be representative of the operational conditions (OC) and risk management measures (RMM) described in the exposure scenario. The task (or combination of tasks) that the dataset represents should be clearly documented, including non-exposure periods. There needs to be sufficient information to satisfactorily support the suitability and representativeness of measurement data. Some of the key requirements on data adequacy set out in [1] are that: sampling and measurement techniques should be appropriate; a clear textual description of activities and monitored tasks should be documented; information on OC and RMM in place at the time of sampling should be provided; the date(s) when sampling was undertaken should be provided (to indicate whether data are current or historic); and summary statistics based upon measurement data should be available. The EN689-2018 European standard [2] (EN689, 2018) covers similar ground for conducting measurement campaigns, although with a focus on establishing workplace compliance with occupational exposure limit values (OELVs) and thus has direct relevance to workplace health and safety.

In terms of a translation to practical guidance for measurement campaigns, [1] notes that at least six measurements are required to adequately quantify the exposures of a single work activity within one company. There is a recognition that exposure assessments for broader exposure scenarios require larger measurement datasets to ensure sufficient coverage and to identify potentially relevant subsets, but this does not neatly translate into hard requirements for numbers of measurements. However, variability and estimated proximity to a risk characterisation ratio (RCR) of unity are noted as considerations when designing a measurement campaign.

The R.14 guidance document [1] further notes that measured datasets with only partial information on context available will usually not be suitable for an assessment under REACH. Although the guidance does contain provisions for read-across from ‘analogous situations’ (for one substance to another or for the use of a substance within a broadly similar work environment), there has been only limited progress into the development of a framework to support read-across in practice [3].

Perhaps unsurprisingly given the cost of taking measurements, the fairly limited support for read-across, the requirement for collaboration over companies and sites, and the fairly opaque guidance on requirements for the design of measurement campaigns, fewer than 5% of registration dossiers submitted under REACH are supported, at least partially, by measurement data (personal communication with Celia Tanarro (ECHA)). Practical exposure assessment under REACH has a heavy reliance on exposure models.

Conceptual models that estimate occupational exposures based upon information about substances and their conditions of use have been under development for almost 30 years, with the first such broad scope model for regulatory risk assessment being the Estimation and Assessment of Substance Exposure (EASE) model [4,5]. There have been substantial improvements in the precision and reliability of more recent exposure models compared to EASE [5]. Exposure models such as the Targeted Risk Assessment (TRA) [6], Stoffenmanager^®^ [7], and the Advanced REACH Tool (ART) [8], are now widely used in regulatory risk assessments for industrial chemicals as well as in the management of these and other processes that generate hazardous substances in workplaces [5]. There has been a recent challenge to the theoretical basis of commonly used exposure models under REACH [9,10] and a robust defence from model authors [5,11]. Recent work suggests that a weighted average of predictions from the TRA, Stoffenmanager^®^ and ART is more reliable than any one model [12].

The Advanced REACH Tool (ART) is the most detailed exposure model currently available for estimating inhalation exposures to dusts, vapours, and aerosols under a broad range of exposure scenarios. A Bayesian statistical model underpins the ART. In the absence of measurements, estimates for a given exposure scenario are derived from the calibrated mechanistic model and information on within-worker, between-worker, and between-company variability from meta-analyses in the literature [13,14]. These information sources specify informative prior distributions for parameters in the statistical exposure model. Quantities of interest such as the geometric mean (GM), which are functions of model parameters, have implied prior distributions. Henceforth, the suite of directly specified and implied prior distributions corresponding to a particular exposure scenario will be referred to as the prior distribution of the ART (specific to a particular exposure scenario). Uniquely amongst the reviewed exposure models, the ART provides a facility to update the baseline estimates using measurement data collected on the exposure scenario. For consistency with [15] and the ART webtool, such data are referred to as being fully analogous. In contrast to the careful consideration of data requirements required for measurement-based submissions, the fully analogous measurement data need not be from a representative sample of companies and workers: the underpinning exposure model of the ART accounts for the context of measurements through company and worker labels and has no lower limit on the number of measurements. However, the technical requirements on data quality still have to be met.

In a recent review, summarise the findings from a decade of validation studies and other applications of the ART model in terms of model reliability and bias are summarised [16]. Strikingly, outside the testing of the Bayesian model [15] (McNally et al., 2014) only a single publication [17] utilised the Bayesian module of the software to update the mechanistic model estimate. This is consistent with user statistics collected from the ART software application, which indicate only a small fraction of visits to the ART homepage result in the Bayesian module being invoked: exposure assessments using the ART are almost entirely based upon the prior distribution of the ART (which is fully defined by user input when constructing an exposure scenario).

In this novel study, the full capability of the ART tool is demonstrated using a small number of carefully chosen case studies. Initially the performance of the Bayesian module of the ART is studied in cases where summary statistics derived from measurement data are consistent with estimates from the prior distribution of the ART. Three case studies are developed to demonstrate how central estimates and uncertainty (represented by the bounds of a 90% credible interval) evolve as the prior distribution of the ART is updated using small measurement datasets. In a further three case studies, the performance of the ART is studied in instances where there is a significant inconsistency between estimates of exposure from the mechanistic model and measurement datasets, respectively (a prior-data conflict). These examples study how the prior is updated as progressively larger measurement datasets are utilised to update the prior. The ultimate aims of this original work are to demonstrate the value of small measurement datasets (of between two and ten measurements) in rapidly reducing the uncertainty in estimates of exposure, and in resolving an inconsistency between the ART mechanistic model and measurement data, thus demonstrating the significant value that is added through utilising even small (and potentially non-representative) measurements datasets.

## 2. Materials and Methods

### 2.1. Overview of Approach

A high-level conceptual overview of the modelling approach is briefly described below with further technical details described in subsequent sub-sections. The aim of this work was to develop a series of case studies, with variations in substance and exposure scenario characteristics, in order to study how estimates from the prior distributions that underpin the ART model updated as progressively larger measurement datasets were utilised.

Two summary statistics were selected for this purpose: the GM and the 90th percentile of exposures. These quantities were initially calculated based upon the ART mechanistic model and variance components. Datasets of between 2 and 10 measurements were subsequently used to update the estimates from the prior. The benchmark for comparisons was the posterior distribution derived using the complete measurement dataset. However, a minor adaption of the ART exposure model was first implemented (as described below) to completely remove the influence of the mechanistic model of the ART. The overall convergence towards this ‘full’ posterior was studied as the size of the measurement dataset increased. Furthermore, the variability in results from different measurement subsets was also studied.

### 2.2. Case Studies

An exposure measurement library comprising of 1944 measurements associated with 117 exposure scenarios was included in version 1.5 of the ART webtool [18]. The exposure scenarios from an augmented dataset (covering 121 exposure scenarios) were recently used in the development of a read-across approach [3]. A summary spreadsheet was available from this work, which contained a textual description of the scenario, an empirically calculated GM, an estimate of the GM from ART, the total number of measurements, and the numbers of unique companies and workers covered by the data, for each exposure scenario. A consistency score (Equation (1)) between ART and empirical estimates of the GM was calculated for each scenario, where $GM_{ART}$ and $GM_{Emp}$ denote ART and empirical estimates of the GM, respectively, and $\theta_{\mu }$ denotes the mechanistic model standard deviation appropriate to the substance class (dust, aerosol, vapour, solid objects) for the particular exposure scenario.

(1) $$\mathrm{Consistency}\;\mathrm{Score}=\left|\frac{\ln\left(GM_{ART}\right)-\ln\left(GM_{Emp}\right)}{\theta_{\mu }}\right|$$

A key requirement in the selection of case studies was that datasets should be of sufficient breadth to support a measurement-data based exposure assessment. For the ‘normal use’ case studies, the three measurement datasets with best measurement coverage (number of measurements and unique companies and workers) were selected from the subset of scenarios where the consistency score (Equation (1)) was less than 2. For the prior-data conflicts, a similar selection was made; however, the appropriate exposure scenario subset was from scenarios where the consistency score (Equation (1)) was greater than 2. This latter subset of case studies was particularly challenging for the ART.

A textual description of each selected exposure scenario (as provided in the ART measurement database) is given below, with summary information of these studies provided in Table 1. ART reports, following coding of these scenarios using the ART webtool, are provided in Supplementary Material.

#### 2.2.1. Case Study (a): Spreading of Glue

This scenario describes the spreading of solvent-containing products during several cobbling processes. Operators were sampled for between 240 and 750 min and used solvents for one to two hours within the measurement period. The products were applied with an application rate of 0.3–1 m^2^/h. Fixed capturing hoods were used for localized control. Various products were used with a mean vapour pressure for analytes within the products of 6900 (range of 20–11,500) Pa. The products used contained a mean of 80% solvents (range 37–100%). The activities were performed in rooms of 50–450 m^3^ with a range of ventilation configurations (without ventilation, mechanical or natural ventilation). The exposure measurements reflect exposure levels to total hydrocarbon vapours.

#### 2.2.2. Case Study (b): Plastering of Walls

The scenario describes the plastering of walls in new buildings and during renovations. Operators were sampled for between 202 and 286 min and were working for the full sampling period. The measured exposure was the result of near field handling of substantially and visibly contaminated objects. No localized controls were provided. The exposure was to a fine powder (plaster dust). The plastering was performed in rooms of 30–1000 m^3^ including both mechanical and natural ventilation. A co-worker was present performing the same task. The exposure measurements reflect exposure to inhalable dust.

#### 2.2.3. Case Study (c): Electroplating

This scenario describes the work of operators in the galvanizing industry. The operators were measured between 40 and 240 min. The operators were exposed to chromium released from galvanizing baths. More than one galvanizing bath (containing chromium) was present in the workplace. The total surface area of galvanizing baths ranged between 0.3 and 1 m^2^. The operators performed manual work and semi-automated processes (using hoists), so both near-field and far-field exposures occurred during the activities. The baths were provided with local exhaust ventilation on the edges of the baths. The concentration of chromium in the baths was approximately 20%. The activities were performed in rooms of 300–3000 m^3^ with either no ventilation or mechanical ventilation. The exposure measurements reflect exposure levels to aerosolised chromium.

#### 2.2.4. Case Study (d): Mixing Drugs

This scenario describes the mixing of drugs in pharmacies resulting in exposure to a coarse dust. Operators were sampled for 50–55 min and were involved in mixing for the entire sampling period. The product used was Pyridoxine. Mixing the product was performed at a use rate of <10 g to 1 kg with careful handling. No localized controls were provided. The activity was performed in a room of 30–100 m^3^ with mechanical ventilation. Demonstrable and effective housekeeping was in practice. The exposure measurements reflect exposure levels to the ingredient in the inhalable dust measured (pyridoxine hydrochloride).

#### 2.2.5. Case Study (e): Sawing of Wood

This scenario describes the work of circular saw operators in wood-working premises. Operators were measured between 233 and 512 min. Operators were exposed to wood dust during the entire measurement period. The task involved directing wood towards the spinning circular saw protruding through the work surface. The task may be classified as mechanical handling of wood resulting in large amounts of dust. The source was in the near-field of the worker. The blade guard partially enclosed the source. LEV was applied through on tool extraction. The activity was performed in large rooms in excess of 3000 m^3^ volume with general or natural ventilation. The exposure measurements reflect exposure to inhalable dust.

#### 2.2.6. Case Study (f): Pumping Gasoline

This scenario describes the work of operators in the car recycling industry. The operators were measured between 15 and 277 min. Operators were exposed to benzene from gasoline during the whole measurement period. Before cars were dismantled, workers drained fuel out of the fuel tanks using a closed system with a pump. The gasoline was pumped away and collected in a vessel. During 50% of the sampling period the worker was located in the near-field of the pump and the remaining time was spent in the vicinity of the pumping process. No localized controls were provided. The gasoline contained approximately 1% benzene. The activity was performed in rooms of 300–3000 m^3^ with natural ventilation. The exposure measurements reflect exposure levels to benzene vapour.

### 2.3. Statistical Modelling

#### 2.3.1. ART Exposure Model

The underlying statistical model of the ART assumes that every relevant exposure scenario has a distinct exposure distribution that is adequately represented by a lognormal mixed effects model, with random-effects representing between-company and between-worker variability, and a residual error representing within-worker variability. The model, as applied to a given exposure scenario of interest, can be written

(2) $$\ln\left(Y_{\mathrm{ijk}}\right)=\mu +c_{i}+w_{ij}+\varepsilon_{ijk}$$

(3) $$c_{i}\sim N\left(0,\;\sigma_{bc}^{2}\right)$$

(4) $$w_{ij}\sim N\left(0,\;\sigma_{bw}^{2}\right)$$

(5) $$\varepsilon_{\mathrm{ijk}}\sim N\left(0,{\;\sigma }_{\mathrm{ww}}^{2}\right)$$

In Equation (2), $Y_{\mathrm{ijk}}$ represents measurement k on worker j within company i. Parameter μ denotes the natural log of the geometric mean exposure associated with the exposure scenario, $\sigma_{bc}$ and $\sigma_{bw}$ denote between-company and between-worker standard deviations, respectively, and $\sigma_{ww}$ represents the within-worker standard deviation.

In practical use of the ART, estimates of exposures are achieved through a two-stage process. In the first stage, estimates are based upon informative prior distributions for model parameters μ (Equation (6)) and the standard deviations $\sigma_{bc}$, $\sigma_{bw}$ and $\sigma_{ww}$, (Equations (7)–(9)), where the hyper-parameters of the priors are based upon the substance class and some characteristics of the exposure scenario (Table 2). Parameter $\bar{\mu }$ in Equation (6) denotes the natural log of the mechanistic model estimate from the ART and is estimated based upon user input [19], whereas $\theta_{\mu }$ characterises uncertainty in the mechanistic model estimate, with substance-class specific standard deviations estimated during model calibration [20].

(6) $$\mu \sim N\left(\bar{\mu },\theta_{\mu }^{2}\right)$$

(7) $$\ln\left(\sigma_{bc}\right)\sim N\left(\ln\left(GM_{bc}\right),\ln\left(GSD_{bc}\right)\right)$$

(8) $$\ln\left(\sigma_{bw}\right)\sim N\left(\ln\left(GM_{bw}\right),\ln\left(GSD_{bw}\right)\right)$$

(9) $$\ln\left(\sigma_{ww}\right)\sim N\left(\ln\left(GM_{ww}\right),\ln\left(GSD_{ww}\right)\right)$$

Based upon the prior specification alone, point-estimates and probability distributions that capture the uncertainty in the model parameters are available. Furthermore, summary statistics based upon the model parameters also have implied prior distributions.

In the (optional) second phase of analysis, estimates for model parameters (and summary statistics calculated from model parameters) are updated based upon personal exposure measurements related to the exposure scenario. Each row of data contains a measurement and company and worker labels so that measurements may be associated with given companies and workers.

In instances where measurement data are used to update the prior distribution of the ART, statistical inference for model parameters is made using Markov Chain Monte Carlo (MCMC). In the ART webtool, the OpenBugs software [21] is used for MCMC sampling.

#### 2.3.2. Initial Runs

The ART webtool was used to code each of the six case studies described above and the model was run. The natural log of the central estimate of the GM was taken from output to provide the central estimate of $\bar{\mu }$. The four standard deviations required for the simulations were based on contextual information relating to substance class and setting (Table 2). A local WinBugs [21] implementation of the statistical model was used to draw samples from the prior distributions of the GM and 90th percentile. The median and a lower (LCL) and upper confidence limit (UCL), corresponding to 5th and 95th percentiles, respectively, were extracted from output on these two summary statistics.

In the second run, all available measurements associated with the exposure scenario were used to update the prior distribution and derive data-led estimates. The prior distribution for μ was adapted in order to completely remove the influence of the mechanistic model estimate through setting $\theta_{\mu }=1000$ in Equation (6); however, the prior distributions for variance components (6–8) were identical under the two runs. The results from this analysis are referred to henceforth as the adapted ART posterior distribution. Data-led estimates for the GM and 90th percentiles, summarised through posterior medians, LCL and UCL were obtained.

#### 2.3.3. Simulation Framework and Analysis

Measurement data subsets were sampled from the full measurement dataset and used to update the prior distributions. One thousand replicates were obtained for data samples of two through to ten measurements. For each replicate, sampling without replacement from the full measurement dataset was used to generate the measurement subset, with each subset differing not only in the measurements themselves, but also in contextual information associated with the measurements (i.e., company and worker labels). For each replicate, the posterior median, LCL and UCL for the GM and 90th percentiles were calculated from the model output and stored.

Two measures were computed from simulation output in order to summarise the results. The first measure focussed on the central estimates of the GM and 90th percentile. The posterior medians from the 1000 simulations at each sample size were ordered by magnitude and the 50th, 500th and 950th values, corresponding to the 5th, 50th and 95th percentiles of ordered output, were extracted. The 50th percentile may be interpreted as the behaviour of the average update at a given sample size. The 5th and 95th percentiles define an interval which contains 90% of the posterior medians corresponding to a given sample size: this 90% interval thus provides information on the variability in the posterior median as a consequence of the composition of the measurement subset. The second measure was the ratio of the UCL to LCL, which characterises the uncertainty associated with estimates of the GM and 90th percentile. This ratio was calculated for each replicate at each sample size. The ratios for 1000 replicates at a given sample size were ordered with the 50th, 500th and 950th values extracted as summaries. From these summaries, the average reduction and variability in the reduction in uncertainty, relative to the prior, could be studied.

A third measure was exclusively used to study the three case studies with prior-data conflicts to assess the variability in convergence as a consequence of updating using different measurement data subsets. This was done through assessing the consistency of results between an update with a given measurement dataset compared with results obtained from the adapted ART posterior. The initial measure of consistency between estimates of the GM and 90th percentile from a given measurement subset and the adapted ART posterior was to ascribe them as being consistent if the two credible intervals overlapped. A second—stronger—measure of consistency was also investigated, whereby consistency was only achieved if the adapted ART posterior median for the summary statistic (GM or 90th percentile) was within the 90% credible interval based on a given measurement dataset.

#### 2.3.4. Computational Framework

The ART webtool was used in order to derive $\bar{\mu }$ since this offers an efficient and robust (to user-error) method for stepping through the underpinning source–receptor model; however, once this estimate was available all simulations were run using a WinBugs implementation of the statistical model. A computationally efficient workflow was devised through interfacing the R software [22] with Winbugs using the R2WinBugs package [23]. Specifically, a script was written in R for sampling a dataset which was passed alongside model parameters to Winbugs using the R2WinBugs package, the simulation was run in Winbugs, and simulation results were returned back into the R operating environment following completion of the simulation. Summary data were extracted from MCMC output and stored. The workflow described above took approximately two seconds for each replicate.

## 3. Results

### 3.1. Cases with Prior-Data Consistency

**Figure 1:**
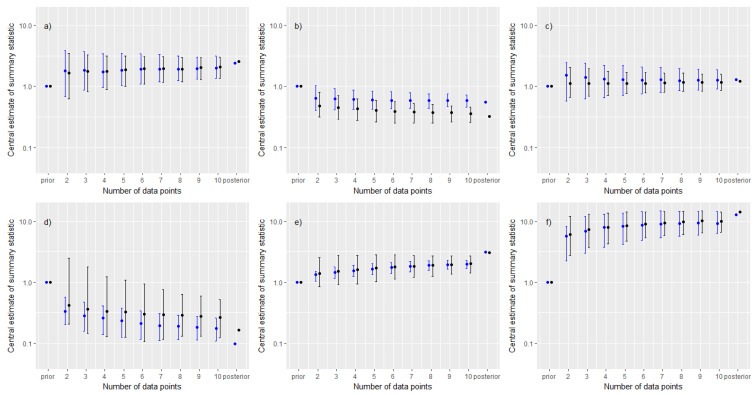
A comparison of posterior medians (blue denotes results for GM, black denotes results for the 90th percentile of exposures) against sample size. Solid points correspond to the 500th value from an ordered series of simulation output at each sample size; interval corresponds to 50th and 950th values. All results normalised relative to the prior medians. Panel labels (**a**–**f**) correspond to case study labels.

**Figure 2:**
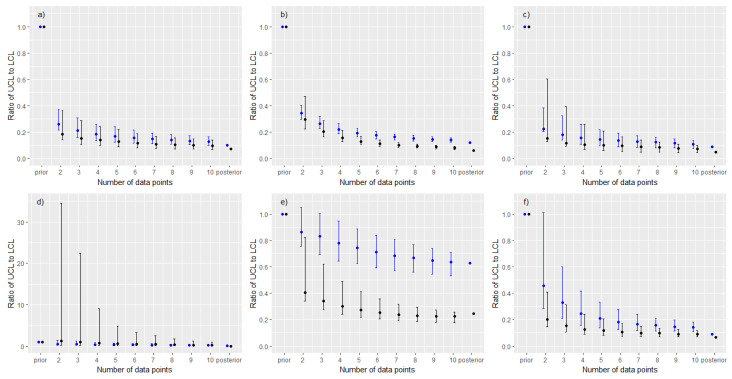
A comparison of the ratio of UCL to LCL (blue denotes results for GM, black denotes results for 90th percentile of exposures) against sample size. Solid points correspond to the 500th value from an ordered series of simulation output at each sample size; interval corresponds to 50th and 950th values. All results normalised relative to ratio of UCL to LCL under the prior. Panel labels (**a**–**f**) correspond to case study labels.

Panels (a) to (c) Figure 1 show the results for the GM and 90th percentile based on posterior medians. In each panel, the series of points plotted for between two and ten measurements correspond to 500th values of the ordered series of posterior medians (computed from the 1000 replicates at that sample size), with the intervals corresponding to the 50th and 950th values. Results from the prior and adapted ART posterior are shown for comparison. To allow for a meaningful comparison over the case studies all the results are normalised with respect to the median values from the priors. Panels (a) to (c) of Figure 2 show the results based upon the ratio of UCL to LCL. In each panel, the points correspond to the 500th value of the ordered series of UCL:LCL ratios, with the intervals corresponding to the 50th and 950th values. Results are all normalised with respect to the ratio of UCL to LCL under the prior.

The appropriate benchmarks for assessing results against are the posterior medians for the GM and 90th percentile corresponding to the adapted ART posterior. Results in panels (a) to (c) of Figure 1 indicate that on average, the posterior median estimates of both of these summaries were very close to the benchmark values when updating the prior with as few as two measurements. However, at lower sample sizes there was considerable variability (represented by the interval) in the posterior median estimates of the GM and 90th percentile, with results proving to be very sensitive to the sampled measurement data subset. Updates with some small measurement subsets resulted in a posterior median that was further away from the adapted ART posterior, relative to the prior—this was most pronounced for the third case study.

Results in panels (a) to (c) of Figure 2 illustrate the reduction in uncertainty associated with estimates of the GM and 90th percentile within the individual simulations. The very rapid reduction in uncertainty after including only a few data points was apparent in all three case studies. Relative uncertainty associated with the 90th percentile decreased at a faster rate; however, this summary was more sensitive to the subset of data used in the update, particularly for very small measurement subsets (Figure 2, panels (a)–(c)). All three case studies showed a very substantial reduction in relative uncertainty with as few as two measurements.

### 3.2. Cases with Prior-Data Conflicts

**Figure 3:**
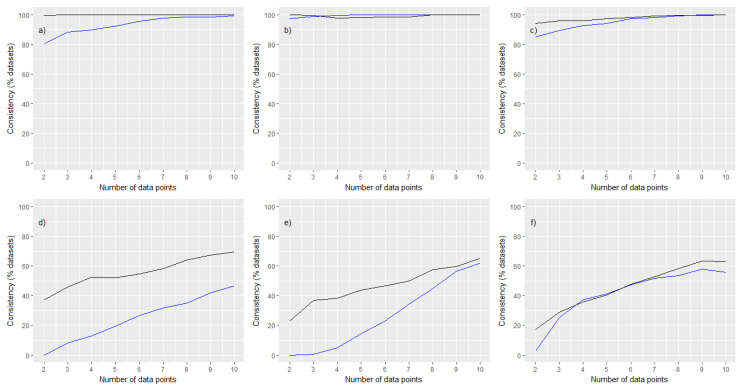
A comparison of consistency in case studies (**a**–**f**) (blue denotes results for GM, black denotes results for 90th percentile of exposures). **Upper** panels show the percentages of simulations where the 90% credible interval resulting from an update with a measurement subset and the adapted ART posterior overlapped. **Lower** panels show the percentage of simulations where the posterior median from the adapted ART posterior was contained within the 90% credible interval resulting from an update with a measurement subset.

Panels (d) to (f) Figure 1 show the results for the GM and 90th percentile based on posterior medians. Panels (d) to (f) of Figure 2 show the results based upon the ratio of UCL to LCL. The percentages of the 1000 simulations at each sample size that resulted in posterior distributions for the GM and 90th percentile that were consistent with those from the adapted ART posterior, under the two definitions of consistency described in methods, are plotted in Figure 3.

One finding that was replicated in each of these latter three case studies was that the estimate of the GM from the prior was rapidly corrected after updating with as few as two measurements, particularly so for case study (f). A further finding over these three case studies was that the weaker measure of consistency (Figure 3, panels (a) to (c)) was quickly satisfied by almost all of the simulations, even at very small measurement subsets. However, the more stringent measure of consistency (Figure 3, panels (d) to (f)) proved more difficult to achieve and generally required larger sample sizes. In general, measurement datasets that covered the widest range of companies resulted in the greatest changes to estimates of GM and 90th percentile, relative to the prior.

In case study (e), results were notably poorer with the posterior medians for the GM and 90th percentile from the adapted ART posterior clearly offset from the posterior medians estimated using measurement data subsets. Furthermore, the uncertainty associated with the estimate of the GM as characterised by the UCL to LCL ratio narrowed very slowly. These results appear to be as a consequence of the much smaller standard deviation $\theta_{\mu }$ associated with a mechanistic model estimate for the abrasion of solid objects (Table 2), which encodes a greater prior-data conflict that is difficult to resolve. The larger values of $\theta_{\mu }$ for dusts and in particular vapours (Table 2) encode a weaker prior-data conflict.

Results were unusual for case study (d), particularly so for the 90th percentile. The ratio of UCL to LCL was large in a subset of the simulations at each sample size, particularly for very small measurement subsets. This behaviour was ultimately traced to updates made with single-worker measurement subsets (i.e., all measurements corresponded to a single worker). In these cases, detailed analysis indicated that the posterior distribution for the between worker variability was more diffuse than the prior, and in particular the upper tail supported very large between-worker standard deviations, which in turn led to a very diffuse posterior distribution for the 90th percentile.

## 4. Discussion

As was set out in the introduction to this study, the opaque requirements for the size of measurement datasets, the requirement for sufficient coverage of sites undertaking similar tasks, and the requirement that data are representative of the OC and RMM in place, present significant barriers to the widespread utilization of measurement datasets to support exposure assessments. The vast majority of occupational exposure assessments under REACH are instead supported by exposure models, principally the tier one ECETOC TRA tool (personal communication with Celia Tanarro (ECHA)).

The Advanced REACH Tool is unique amongst the available exposure models in that it supports a hybrid model-measurement approach through a Bayesian exposure model. In the absence of measurement data, inference solely from the mechanistic model and variance components is possible. If there are supporting measurement data, there is no requirement for a minimum number of measurements; nor do the supporting fully analogous measurement data need to be from a representative subset of workers and companies because the hierarchical exposure model accounts for the context of exposure measurements. However, user statistics from the ART webtool indicate the facility of the software to incorporate analogous personal exposure measurement data is little used. The ultimate aim of this research was to demonstrate the value of small measurement datasets of between two and ten measurements in both refining estimates from the prior and in reducing the uncertainty associated with key summaries. Results from this work suggest that by encouraging users to make better use of the software capability, even when only few measurements are available, more precise and reliable estimates from the ART can be obtained. Bespoke industry wide measurement campaigns are not necessary; data from routine sampling suffice, although there are clearly requirements on technical adequacy, etc., to be satisfied which are as stringent as under the REACH regulations.

It is important to stress that the conflicts observed in the latter three case studies in this work are not representative of the expected performance of the ART. These case studies were ‘cherry picked’ to provide a challenge for a range of substance classes (and hence different prior distributions) and had sufficient measurement data available to support a measurement-based exposure assessment. In practical assessment, discrepancies between estimates from the ART mechanistic model and measurement data may arise as a consequence of two principal reasons. Firstly, variability in personal exposures may be significant: even for the case studies in this work, where measurement data were of sufficient breadth to support a measurement-based exposure assessment, the uncertainty in the GM and 90th percentile, as characterised by the width of the credible interval from the adapted ART posterior, is non-trivial. For the typically much smaller datasets that have been used in some validation studies, the uncertainty associated with measurement-based estimates of the GM and 90th percentile is significantly greater. Clearly uncertainty in both measurement-based and model-based estimates should be considered in thorough validation studies. Secondly, there is model error. There are three subclassifications of model error: (a) conceptual error in the exposure model; (b) the authors’ conception of the exposure scenario is erroneous; and (c) the exposure scenario is incorrectly transcribed into the determinants of the model. Errors of these latter two classes result in a measurement dataset that is not fully analogous to the coded exposure scenario. The case studies in this work cover all three classes of model error: the conflict in the first case study appears to result from model error; the second case study represents an error in the assessor’s conceptualisation of the scenario (Subsequent to scoring this scenario the visiting occupational hygienist was consulted. The scoring of the primary task was judged to be sound; however, a second activity involving the handling of contaminated objects, resulting in exposure to a fine dust, had not been accounted for. This latter near field exposure for a small fraction of the measurement period is likely to have dominated the exposure.); the conflict in the third case study likely results from an incorrect assignment for the level (scored medium) of containment.

Inter-individual variability in the scoring of exposure scenarios has been previously studied [24] and found to be substantial. As far as the author is aware, the impact of the assessor’s misconception of exposure scenarios, where sub-tasks leading to significant exposures are not characterised, has not been previously considered. Both of these sources of model error are likely to be significant for exposure scenarios coded for the purposes of validation from literature sources and, without recourse to original data nor expertise, should be explicitly considered when evaluating model performance in the context of validation studies.

Our final comments relate to the resolution of prior-data conflicts. Results from simulations (Figure 1d–f and Figure 3) demonstrated that measurement data from a cross-section of workers and companies offered the best breadth of data with which to improve estimates. However, whilst the updates with measurement subsets moved towards the adapted ART posterior as greater numbers of measurements were used, the prior-data conflict never fully resolved (as seen by the central estimates sitting consistently above/below the corresponding values from the adapted ART posterior), with the posterior converging to the region of parameter space that minimised the prior-data conflict.

The current form of the ART exposure model is not formulated to efficiently resolve prior-data conflicts; however, there is a rich literature [25,26,27,28,29,30] to inform suitable adaptions to Equations (6)–(9). Specifically, adapting the prior for μ by using a heavier tailed distribution compared with the current Gaussian may facilitate the more rapid resolution of prior-data conflicts. A t-distribution with low degrees of freedom might represent one viable alternative for modelling uncertainty in the mechanistic model estimate. Whilst the remedy described above would result in a technically correct resolution, this would be achieved through a ‘rejection’ of the prior in favour of measurement data. It is not clear this is desirable without first providing feedback to an assessor that the ART mechanistic model appeared to be inconsistent with measurements: awareness of this conflict may prompt a re-evaluation of assessments or further investigations as to whether the data were fully analogous as previously assumed. The results from case study (d) suggest that under certain conditions a significant prior-data conflict might be identified with as few as two measurements (in these special cases the ratio of the UCL to LCL massively increased compared to the prior). Ongoing work is attempting to establish whether the results from this case study might inform an efficient screening methodology for rapidly identifying and communicating such conflicts based upon ART output.

## 5. Conclusions

The results from this simulation study suggest that small measurement datasets have the potential to substantially reduce the uncertainty associated with estimates of the GM and 90th percentile, particularly when the measurements are sourced from a representative subset of workers and companies associated with the exposure scenario. Furthermore, very rapid improvements in a poor estimate from the ART mechanistic model may be achieved with as few as two or three measurements. Further research is required for rapidly identifying prior-data conflicts. A conceptual workflow for communicating and resolving prior-data conflicts needs to be developed and tested prior to considering an alternative robust prior specification in the ART webtool.

## Figures and Tables

**Table 1:** Summary data on the six case studies.

Scenario	Activity Class	Substance Class	Setting	Measurements, Companies, Workers		
Spreading glue	Spreading of liquid products	Vapour	Indoors	85	14	14
Plastering walls	Handling of contaminated solid objects or paste	Dust	Indoors	21	7	21
Electroplating	Activities with agitated surfaces	Aerosol	Indoors	24	6	24
Mixing drugs	Movement and agitation of powders, granules and pelletised products	Dust	Indoors	41	9	13
Sawing wood	Fracturing and abrasion of solid objects	Solid object	Indoors	17	7	17
Pumping gasoline	Falling liquids	Vapour	Indoors	36	7	36

**Table 2:** Hyper-parameters of the ART mechanistic model for four different substance classifications and two exposure scenario settings.

Substance Class	Setting	$\theta_{\mu }$ ^1^	$\sigma_{bc}$ ^2^		$\sigma_{bw}$		$\sigma_{ww}$	
			GM	GSD	GM	GSD	GM	GSD
Dusts	Indoors	0.89	0.44	1.29	0.32	2.82	0.65	1.64
	Outdoors	0.89	0.44	1.29	0.32	2.82	1.57	1.64
Vapours	Indoors	0.97	0.44	1.29	0.26	2.82	0.48	1.64
	Outdoors	0.97	0.44	1.29	0.26	2.82	1.16	1.64
Mists (low-volatiles)	Indoors	1.06	0.44	1.29	0.32	2.82	0.65	1.64
	Outdoors	1.06	0.44	1.29	0.32	2.82	1.57	1.64
Solid object/abraision	Indoors	0.46	0.44	1.29	0.32	2.82	0.65	1.64
	Outdoors	0.46	0.44	1.29	0.32	2.82	1.57	1.64

^1^ This parameter denotes the standard deviation associated with the mechanistic model estimate. ^2^ The GM and GSD parameterise log-normal distributions for between-company, between-worker and within-worker sources of variability.

## Data Availability

Not applicable.

## Supplementary Materials

The following supporting information can be downloaded at: https://www.mdpi.com/article/10.3390/ijerph20075386/s1, ART reports for case studies 1–6.
