# Supplementary material for: How Valuable Are Small Measurement Datasets in Supplementing Occupational Exposure Models? A Numerical Study Using the Advanced Reach Tool
Source: Int J Environ Res Public Health. 2023 Apr 4;20(7):5386. doi: 10.3390/ijerph20075386 (PMC10094536; doi:10.3390/ijerph20075386)
Supplement: Supplementary file 1 [file ijerph-20-05386-s001.zip › ijerph-2256532-supplementary.pdf]

ART REPORT – Case study a): Spreading of glue – 19-Jan-23

This scenario describes the spreading of solvent-containing products during several cobbling processes. Operators were sampled for between 240 and 750 minutes and used solvents for one to two hours within the measurement period. The products were applied with an application rate of 0.3-1 m2/hour. Fixed capturing hoods were used for localized control. Various products were used with a mean vapour pressure for analytes within the products of 6900 (range of 20-11500) Pa. The products used contained a mean of 80% solvents (range 37-100%). The activities were performed in rooms of 50-450 m3 with a range of ventilation configurations (without ventilation, mechanical or natural ventilation). The exposure measurements reflect exposure levels to total hydrocarbon vapours.

Chemical details	
Chemical	Mixed solvents
CAS No.	(unknown)

Scenario details	
Number of activities	1
Total duration (mins)	480
Nonexposure period (mins)	390

Metadata	
ART version	1.5
Creator	kevin.mcnally@hsl.gsi.gov.uk
Date created	03-Nov-22
Date last edited	03-Nov-22

Details for Activity Spreading of glue

Emission sources:	Near field ✓	Duration (mins):	90
	Far field		

Near-field exposure

Operational Conditions

Substance emission potential	
Substance product type	Liquids
Process temperature	Room temperature
Vapour pressure	6900 Pa
Liquid mole fraction	0.8
Activity coefficient	1

Activity emission potential	
Activity class	Spreading of liquid products
Situation	Spreading of liquids at surfaces or work pieces 0.3 - 1.0 m^2 / hour

Surface contamination	
Process fully enclosed?	No
Effective housekeeping practices in place?	No
General housekeeping practices in place?	Yes

Dispersion	
Work area	Indoors
Room size	300 m^3

Risk Management Measures

Localised controls	
Primary	Fixed capturing hood (90.00 % reduction)
Secondary	No localized controls (0.00 % reduction)

Dispersion	
Ventilation rate	Only good natural ventilation

Predicted exposure levels

ART predicts air concentrations in a worker's personal breathing zone outside of any Respiratory Protection Equipment (RPE). The use of RPE must be considered separately.

Mechanistic model results

The predicted 50th percentile full-shift exposure is 11 mg/m^3.

The 90% confidence interval is 2.1 mg/m^3 to 52 mg/m^3.

ART REPORT – Case study b): Plastering of walls – 19-Jan-23

The scenario describes the plastering of walls in new buildings and during renovations. Operators were sampled for between 202 and 286 minutes and were working for the full sampling period. The measured exposure was the result of near field handling of substantially and visibly contaminated objects. No localized controls were provided. The exposure was to a fine powder (plaster dust). The plastering was performed in rooms of 30-1000 m3 including both mechanical and natural ventilation. A co-worker was present performing the same task. The exposure measurements reflect exposure to inhalable dust.

Chemical details	
Chemical	Plaster dust
CAS No.	(unknown)

Scenario details	
Number of activities	1
Total duration (mins)	480
Nonexposure period (mins)	0

Metadata	
ART version	1.5
Creator	kevin.mcnally@hsl.gsi.gov.uk
Date created	03-Nov-22
Date last edited	29-Nov-22

Details for Activity Plastering

Emission sources: Near field ✓ Far field ✓

Duration (mins): 480

Near-field exposure

Operational Conditions

Substance emission potential	
Substance product type	Powders, granules or pelletised material
Dustiness	Fine dust
Moisture content	Dry product (< 5 % moisture content)
Powder weight fraction	1

Activity emission potential	
Activity class	Handling of contaminated solid objects or paste
Situation	Handling of substantially and visibly contaminated objects (layers of more than 0.5 kg).
Handling type	Normal handling, involves regular work procedures.

Surface contamination	
Process fully enclosed?	No
Effective housekeeping practices in place?	No
General housekeeping practices in place?	Yes

Dispersion	
Work area	Indoors
Room size	100 m^3

Risk Management Measures

Localised controls	
Primary	No localized controls (0.00 % reduction)
Secondary	No localized controls (0.00 % reduction)

Dispersion	
Ventilation rate	Only good natural ventilation

Far-field exposure

Operational Conditions

Substance emission potential	
Substance product type	Powders, granules or pelletised material
Dustiness	Fine dust
Moisture content	Dry product (< 5 % moisture content)
Powder weight fraction	1

Activity emission potential	
Activity class	Handling of contaminated solid objects or paste
Situation	Handling of substantially and visibly contaminated objects (layers of more than 0.5 kg).
Handling type	Normal handling, involves regular work procedures.

Risk Management Measures

Localised controls	
Primary	No localized controls (0.00 % reduction)
Secondary	No localized controls (0.00 % reduction)

Segregation	No segregation (0.00 % reduction)

Predicted exposure levels

ART predicts air concentrations in a worker's personal breathing zone outside of any Respiratory Protection Equipment (RPE). The use of RPE must be considered separately.

Mechanistic model results

The predicted 50th percentile full-shift exposure is 18 mg/m^3.

The 90% confidence interval is 4.2 mg/m^3 to 78 mg/m^3.

ART REPORT – Case study c): Electroplating – 19-Jan-23

This scenario describes the work of operators in the galvanizing industry. The operators were measured between 40 and 240 minutes. The operators were exposed to chromium released from galvanizing baths. More than one galvanizing bath (containing chromium) was present in the workplace. The total surface area of galvanizing baths ranged between 0.3 and 1 m2. The operators performed manual work and semi-automated processes (using hoists), so both near-field and far-field exposures occurred during the activities. The baths were provided with local exhaust ventilation on the edges of the baths. The concentration of chromium in the baths was approximately 20%. The activities were performed in rooms of 300-3000 m3 with either no ventilation or mechanical ventilation. The exposure measurements reflect exposure levels to aerosolised chromium.

Chemical details	
Chemical	Chromium
CAS No.	7440-47-3

Scenario details	
Number of activities	1
Total duration (mins)	480
Nonexposure period (mins)	0

Metadata	
ART version	1.5
Creator	kevin.mcnally@hsl.gsi.gov.uk
Date created	03-Nov-22
Date last edited	03-Nov-22

Details for Activity Electroplating

Emission sources: Near field ✓ Far field ✓

Duration (mins): 480

Near-field exposure

Operational Conditions

Substance emission potential	
Substance product type	Powders dissolved in a liquid or incorporated in a liquid matrix
Liquid matrix weight fraction	0.2
Viscosity	Low

Activity emission potential	
Activity class	Activities with agitated surfaces
Situation	Open surface 0.3 - 1 m^2

Surface contamination	
Process fully enclosed?	No
Effective housekeeping practices in place?	No
General housekeeping practices in place?	Yes

Dispersion	
Work area	Indoors
Room size	1000 m^3

Risk Management Measures

Localised controls	
Primary	Fixed capturing hood (90.00 % reduction)
Secondary	No localized controls (0.00 % reduction)

Dispersion	
Ventilation rate	Only good natural ventilation

Far-field exposure

Operational Conditions

Substance emission potential	
Substance product type	Powders dissolved in a liquid or incorporated in a liquid matrix
Liquid matrix weight fraction	0.2
Viscosity	Low

Activity emission potential	
Activity class	Activities with agitated surfaces
Situation	Open surface 0.3 - 1 m²

Risk Management Measures

Localised controls	
Primary	Fixed capturing hood (90.00 % reduction)
Secondary	No localized controls (0.00 % reduction)

Segregation	No segregation (0.00 % reduction)

Predicted exposure levels

ART predicts air concentrations in a worker's personal breathing zone outside of any Respiratory Protection Equipment (RPE). The use of RPE must be considered separately.

Mechanistic model results

The predicted 50th percentile full-shift exposure is 0.0053 mg/m^3.

The 90% confidence interval is 0.00093 mg/m^3 to 0.03 mg/m^3.

ART REPORT – Case study d): Mixing drugs – 19-Jan-23

This scenario describes the mixing of drugs in pharmacies resulting in a coarse dust. Operators were sampled for 50-55 minutes and were involved in mixing for the entire sampling period. The product used was Pyridoxine. Mixing the product was performed at a use rate of <10 g to 1kg with careful handling. No localized controls were provided. The activity was performed in a room of 30-100 m3 with mechanical ventilation. Demonstrable and effective housekeeping was in practice. The exposure measurements reflect exposure levels to the ingredient in the inhalable dust measured (pyridoxine hydrochloride).

Chemical details	
Chemical	Pyridoxine hydrochloride
CAS No.	58-56-0

Scenario details	
Number of activities	1
Total duration (mins)	480
Nonexposure period (mins)	0

Metadata	
ART version	1.5
Creator	kevin.mcnally@hsl.gsi.gov.uk
Date created	03-Nov-22
Date last edited	03-Nov-22

Details for Activity (untitled)

Emission sources:	Near field ✓	Duration (mins):	480
	Far field		

Near-field exposure

Operational Conditions

Substance emission potential	
Substance product type	Powders, granules or pelletised material
Dustiness	Coarse dust
Moisture content	Dry product (< 5 % moisture content)
Powder weight fraction	Pure material

Activity emission potential	
Activity class	Movement and agitation of powders, granules or pelletised material
Situation	Movement and agitation of 0.1 - 1 kg
Agitation level	Handling with low level of agitation
Containment level	Open process

Surface contamination	
Process fully enclosed?	No
Effective housekeeping practices in place?	Yes

Dispersion	
Work area	Indoors
Room size	100 m^3

Risk Management Measures

Localised controls	
Primary	No localized controls (0.00 % reduction)
Secondary	No localized controls (0.00 % reduction)

Dispersion	
Ventilation rate	3 air changes per hour (ACH)

Predicted exposure levels

ART predicts air concentrations in a worker's personal breathing zone outside of any Respiratory Protection Equipment (RPE). The use of RPE must be considered separately.

Mechanistic model results

The predicted 50th percentile full-shift exposure is 0.79 mg/m^3.

The 90% confidence interval is 0.18 mg/m^3 to 3.4 mg/m^3.

ART REPORT – Case study e): Sawing of wood – 19-Jan-23

This scenario describes the work of circular saw operators in wood-working premises. Operators were measured between 233 and 512 minutes. Operators were exposed to wood dust during the entire measurement period. The task involved directing wood towards the spinning circular saw protruding through the work surface. The task may be classified as mechanical handling of wood resulting in large amounts of dust. The source was in the near-field of the worker. The blade guard partially enclosed the source. LEV was applied through on tool extraction. The activity was performed in large rooms in excess of 3000m3 volume with general or natural ventilation. The exposure measurements reflect exposure to inhalable dust.

Chemical details	
Chemical	Wood dust
CAS No.	(unknown)

Scenario details	
Number of activities	1
Total duration (mins)	480
Nonexposure period (mins)	0

Metadata	
ART version	1.5
Creator	kevin.mcnally@hsl.gsi.gov.uk
Date created	03-Nov-22
Date last edited	03-Nov-22

Details for Activity Sawing of wood

Emission sources: Near field ✓ Far field

Duration (mins): 480

Near-field exposure

Operational Conditions

Substance emission potential	
Substance product type	Solid objects
Solid weight fraction	Pure material
Solid material	Wood
Moisture content	Dry product (< 5 % moisture content)

Activity emission potential	
Activity class	Fracturing and abrasion of solid objects
Situation	Mechanical handling of wood resulting in large amounts of dust (e.g., large speed of moving work pieces or rotating cutting blades)
Containment level	Handling that reduces contact between product and adjacent air. Note: This does not include processes that are fully contained by localised controls (see next questions).

Surface contamination	
Process fully enclosed?	No
Effective housekeeping practices in place?	No
General housekeeping practices in place?	No

Dispersion	
Work area	Indoors
Room size	3000 m^3

Risk Management Measures

Localised controls	
Primary	On-tool extraction (90.00 % reduction)
Secondary	No localized controls (0.00 % reduction)

Dispersion	
Ventilation rate	Only good natural ventilation

Predicted exposure levels

ART predicts air concentrations in a worker's personal breathing zone outside of any Respiratory Protection Equipment (RPE). The use of RPE must be considered separately.

Mechanistic model results

The predicted 50th percentile full-shift exposure is 0.37 mg/m^3.

The 90% confidence interval is 0.17 mg/m^3 to 0.79 mg/m^3.

ART REPORT – Case study f): Pumping gasoline – 19-Jan-23

This scenario describes the work of operators in the car recycling industry. The operators were measured between 15 and 277 minutes. Operators were exposed to benzene from gasoline during the whole measurement period. Before cars were dismantled, workers drained fuel out of the fuel tanks using a closed system with a pump. The gasoline was pumped away and collected in a vessel. During 50% of the sampling period the worker was located in the near-field of the pump and the remaining time was spent in the vicinity of the pumping process. No localized controls were provided. The gasoline contained approximately 1% benzene. The activity was performed in rooms of 300-3000 m3 with natural ventilation. The exposure measurements reflect exposure levels to benzene vapour.

Chemical details	
Chemical	Benzene
CAS No.	71-43-2

Scenario details	
Number of activities	2
Total duration (mins)	480
Nonexposure period (mins)	0

Metadata	
ART version	1.5
Creator	kevin.mcnally@hsl.gsi.gov.uk
Date created	03-Nov-22
Date last edited	03-Nov-22

Details for Activity Benzene near and far-field

Emission sources:	Near field ✓	Duration (mins):	240
	Far field ✓		

Near-field exposure

Operational Conditions

Substance emission potential	
Substance product type	Liquids
Process temperature	Room temperature
Vapour pressure	13330 Pa
Liquid mole fraction	0.01
Activity coefficient	1

Activity emission potential	
Activity class	Falling liquids
Situation	Transfer of liquid product with flow of 1 - 10 l/minute
Containment level	Open process
Loading type	Splash loading, where the liquid dispenser remains at the top of the reservoir and the liquid splashes freely

Surface contamination	
Process fully enclosed?	No
Effective housekeeping practices in place?	No
General housekeeping practices in place?	No

Dispersion	
Work area	Indoors
Room size	1000 m^3

Risk Management Measures

Localised controls	
Primary	Medium level containment (99.00 % reduction)
Secondary	No localized controls (0.00 % reduction)

Dispersion	
Ventilation rate	Only good natural ventilation

Far-field exposure

Operational Conditions

Substance emission potential	
Substance product type	Liquids
Process temperature	Room temperature
Vapour pressure	13330 Pa
Liquid mole fraction	0.01
Activity coefficient	1

Activity emission potential	
Activity class	Falling liquids
Situation	Transfer of liquid product with flow of 1 - 10 l/minute
Containment level	Open process
Loading type	Splash loading, where the liquid dispenser remains at the top of the reservoir and the liquid splashes freely

Risk Management Measures

Localised controls	
Primary	Medium level containment (99.00 % reduction)
Secondary	No localized controls (0.00 % reduction)

Segregation	No segregation (0.00 % reduction)

Details for Activity Benzene far-field

Emission sources:	Near field	Duration (mins):	240
	Far field ✓		

Far-field exposure

Operational Conditions

Substance emission potential	
Substance product type	Liquids
Process temperature	Room temperature
Vapour pressure	13330 Pa
Liquid mole fraction	0.01
Activity coefficient	1

Activity emission potential	
Activity class	Bottom loading
Situation	Transfer of liquid product with flow of 1 - 10 l/minute

Surface contamination	
Process fully enclosed?	No
Effective housekeeping practices in place?	No
General housekeeping practices in place?	No

Dispersion	
Work area	Indoors
Room size	1000 m^3

Risk Management Measures

Localised controls	
Primary	Medium level containment (99.00 % reduction)
Secondary	No localized controls (0.00 % reduction)
Segregation	No segregation (0.00 % reduction)
Personal enclosure	No personal enclosure (0.00 % reduction)

Dispersion	
Ventilation rate	Only good natural ventilation

Predicted exposure levels

ART predicts air concentrations in a worker's personal breathing zone outside of any Respiratory Protection Equipment (RPE). The use of RPE must be considered separately.

Mechanistic model results

The predicted 50th percentile full-shift exposure is 0.017 mg/m^3.

The 90% confidence interval is 0.0034 mg/m^3 to 0.084 mg/m^3.
